# Impact of comorbid burden on global left cardiac function and prediction models for myocardial function damage: a cardiac magnetic resonance feature-tracking study

**DOI:** 10.3389/fmed.2025.1525334

**Published:** 2025-02-13

**Authors:** XiaoFeng Qu, Miaomiao Bai, Jianbo Lyu, Lili Yin, Jiahui Zhang, Endong Zhao, Lingjun Mei

**Affiliations:** ^1^Department of Radiology, The Second Hospital of Dalian Medical University, Dalian, China; ^2^Department of International Medical, The Second Hospital of Dalian Medical University, Dalian, China

**Keywords:** CMR-FT, comorbid burden, myocardial function damage, myocardial strain, hypertension, type 2 diabetes mellitus, dyslipidemia

## Abstract

**Objective:**

This study aimed to explore the effects of comorbid burden on left cardiac myocardial function in patients without organic heart disease and to construct prediction models for myocardial function damage.

**Methods:**

A total of 82 healthy individuals and 198 patients with comorbid burden who had normal left ventricular ejection fraction (LVEF) were recruited. Comorbid burden included hypertension, type 2 diabetes mellitus (T2DM), and dyslipidemia. Based on the number of comorbidities, the patients were divided into two groups: comorbid burden <2 and comorbid burden ≥2. Cardiac magnetic resonance feature tracking (CMR-FT) was used to measure myocardial strain parameters.

**Results:**

After adjustment, the left atrial (LA) reservoir strain (*p* = 0.011) and conduit strain (*p* < 0.001) were significantly lower in patients with a comorbid burden ≥2. The left ventricular (LV) global longitudinal strain (*p* < 0.001) and global radial strain (*p* = 0.010) were decreased in both the comorbid burden<2 and comorbid burden≥2 groups. The LV global circumferential strain (*p* = 0.006) was reduced in the comorbid burder≥2 group. Comorbid burden combined with male sex, postprandial blood glucose (PBG), and fasting blood glucose (FBG) proved to be excellent predictors of LV myocardial function damage (AUC = 0.848). In contrast, comorbid burden combined with male sex was only a fair predictor of LA myocardial function damage (AUC = 0.651).

**Conclusion:**

CMR-FT can detect left-sided myocardial function damage in patients with comorbid burden but without organic heart disease prior to a decrease in LVEF. Comorbid burden combined with male sex, PBG, and FBG showed excellent predictive ability for LV myocardial function damage. Comorbid burden combined with the male sex showed a fair predictive ability for LA myocardial function damage.

## 1 Introduction

Comorbid burden is present across all ages, especially in older individuals with cardiovascular disease (CVD), and affects the overall prognosis and therapeutic measures in patients with CVD (1). Hypertension, type 2 diabetes mellitus (T2DM), and dyslipidemia are the most common comorbid conditions in patients with CVD and are also the three main risk factors for CVD (2). With disease progression, these factors are prone to induce cardiac structural and functional impairment, leading to a range of cardiovascular complications (1, 2).

Left atrial (LA) dysfunction is considered a significant risk factor for CVD and is independently related to an increased risk of morbidity and mortality (3, 4). Recent research has indicated that LA dysfunction may precede left ventricular (LV) diastolic dysfunction (4). The left atrial volume index (LAVI) is a diagnostic and grading indicator of LV diastolic dysfunction, and the maximum LA volume (LAV _max_) has emerged as an important biomarker for adverse cardiac events. However, the deterioration of left atrial function precedes structural changes (5). Impaired LV function is an independent predictor of major adverse cardiac events, such as heart failure (HF) and sudden death (6). However, global measures such as left ventricular ejection fraction (LVEF) describe a relative volume change and are not sensitive enough to detect subtle changes in the early stages of LV functional impairment (7).

The myocardial strain is defined as the degree of deformation of a myocardial segment from its initial length (L0, usually at end-diastole) to its maximum length (L1, usually at end-systole), and it is expressed as a percentage (8). Assessment of myocardial deformation can indicate early myocardial function impairment in the LA and LV in various heart diseases, including ischemic cardiomyopathy, non-ischemic cardiomyopathy, and LVEF-preserved HF (9). Recently, speckle tracking echocardiography (STE) and cardiac magnetic resonance feature tracking (CMR-FT) have emerged as commonly used strain imaging techniques for the non-invasive assessment of cardiac deformation. However, echocardiography is limited by poor image quality in cases of inadequate echogenic windows, ultrasound dropouts, and reverberations. In addition, assessing the LA strain using STE can be challenging due to the thin atrial wall, LA appendage, and the presence of pulmonary veins (4, 7). CMR-FT is a post-processing technique based on balanced standard steady-state free precession (b-SSFP) sequences. It offers the advantages of high spatial resolution, no additional acquisition sequences, short post-processing time, and no anatomical plane restrictions (7, 8). Therefore, CMR-FT-derived strain parameters are increasingly used to quantitatively assess subclinical myocardial dysfunction (7).

Various studies have explored the relevant mechanisms underlying the effects of hypertension, T2DM, or dyslipidemia on LA or LV structure and function separately (10–12). However, the effect of comorbid burden on the left cardiac system in patients without organic heart disease is not well characterized. Therefore, the purpose of this study was to evaluate the effects of comorbid burden on left cardiac system myocardial function in patients without organic heart disease using CMR-FT-derived LA and LV myocardial strain parameters.

## 2 Materials and methods

### 2.1 Study population

**Figure 1:**
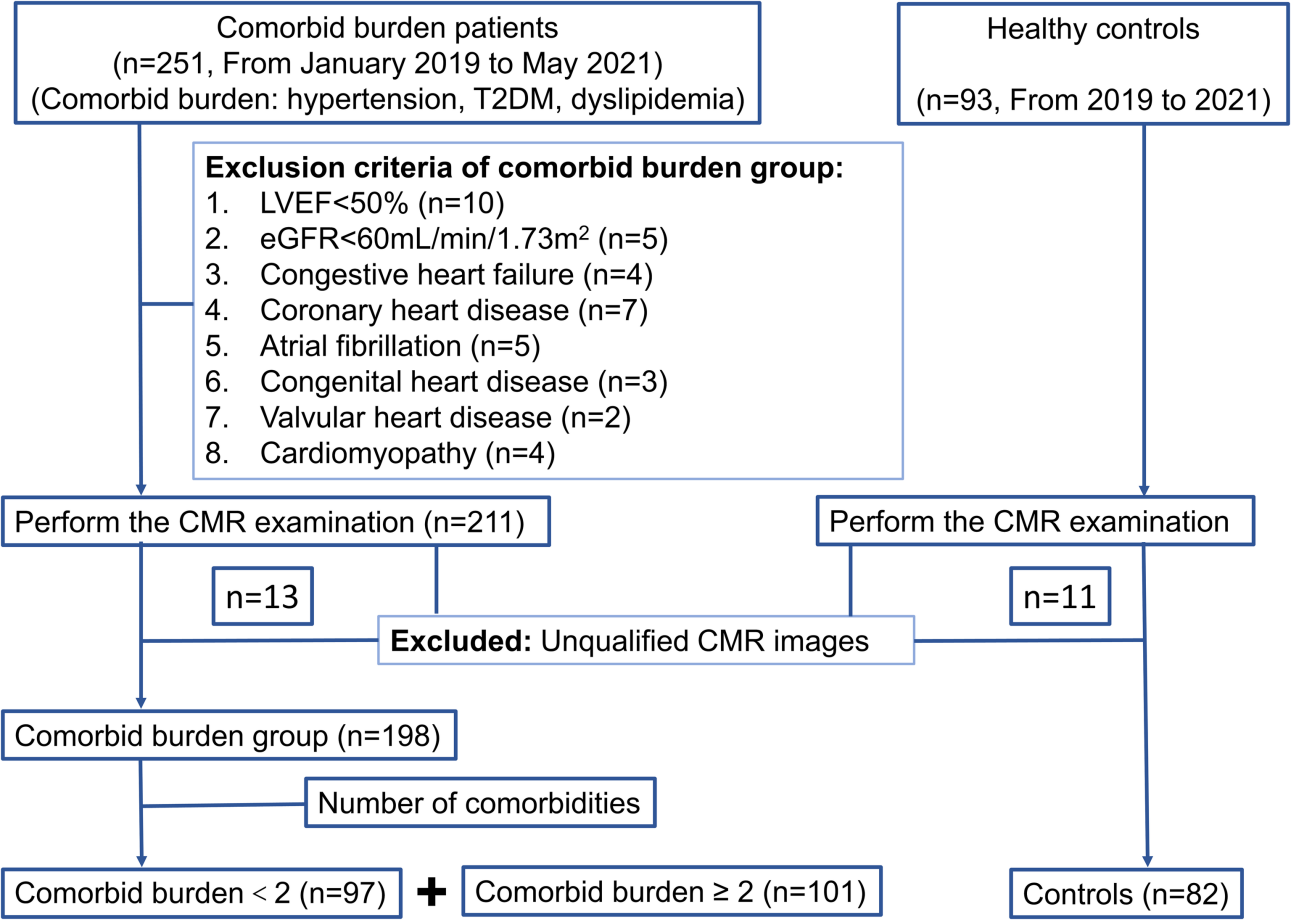
Flow diagram of the cohort study.

A total of 251 patients with comorbid burden and 93 healthy controls were enrolled from January 2019 to May 2021 in this retrospective study. All the participants underwent the same CMR examination on a 3 T scanner. Hypertension was defined as systolic blood pressure (SBP) ≥ 140 mmHg and/or diastolic blood pressure (DBP) ≥ 90 mmHg at rest, measured on more than two occasions, or a history of antihypertensive medication use. The diagnostic criteria for T2DM were based on the current American Diabetes Association guidelines (13). Dyslipidemia included one or more of the following: increased total cholesterol (TC) (≥6.20 mmol/L), low-density lipoprotein cholesterol (LDL-C) (>4.13 mmol/L), and triglyceride (TG) levels (>2.25 mmol/L) or decreased high-density lipoprotein cholesterol (HDL-C) (<1.03 mmol/L) (14). The exclusion criteria were as follows: (1) LVEF<50% (*n* = 10); (2) the estimated glomerular filtration rate (eGFR) <60 mL/min/1.73m^2^ (*n* = 5); and (3) the presence of organic heart disease, such as congestive heart failure, coronary heart disease, atrial fibrillation, congenital heart disease, valvular heart disease, and cardiomyopathy (*n* = 25). In addition, 13 patients with comorbid burden and 11 healthy controls were excluded because of unqualified CMR images (Figure 1).

Finally, 198 patients with comorbid burden were included and further divided into two groups: comorbid burden <2 group (*n* = 97) and comorbid burden ≥2 group (*n* = 101). The comorbid burden <2 group included simple hypertension, T2DM, or dyslipidemia. The comorbid burden ≥2 group included a combination of all the above diseases. A total of 82 healthy volunteers with normal associated examination (clinical presentation, laboratory tests, and imaging examination) but without organic heart disease were included as the control group.

This study was approved by the Clinical Trials and Biomedical Ethics Committee and adhered to the principles outlined in the Declaration of Helsinki. Written informed consent was obtained from all participants.

### 2.2 CMR protocol

All participants were examined using Siemens 3.0 T MRI (Trio Tim; Siemens Medical Solutions, Erlangen, Germany). The b-SSFP cine sequence (repetition time = 3.4 ms; echo time = 1.31 ms; flip angle = 39°; slice thickness = 8 mm; matrix size = 208*139; field of view = 234 mm*280 mm) was performed from the base to the apex level on short-axis and long-axis views (two-chamber, three-chamber, and four-chamber) for continuous cine imaging and subsequent post-processing.

### 2.3 CMR feature tracking

Two radiologists with more than 3 years of CMR experience, who were blinded to the clinical data, evaluated the offline images of all participants using commercial software (cvi42; Circle Cardiovascular Imaging Inc., Calgary, Alberta, Canada).

The short-axis and long-axis sequences (including two-chamber and four-chamber views) were loaded into cvi42 short-axis 3D and bi-planar modules. LA and LV endocardial and epicardial boundaries were delineated semi-automatically at end-diastole. Further manual adjustments were made according to the actual requirements. Then, the minimum LA volume (LAV _min_), LAV _max_, LV mass (LVM) at end-diastole, left ventricular end-diastolic volume (LVEDV), left ventricular end-systolic volume (LVESV), and LVEF were computed automatically. The LA active emptying fraction (LAEF) was calculated as (LAV _max_ – LAV _min_)/ LAV _max_*100%. The LAVI was calculated as LAV _max_/body surface area (BSA). The LV mass index (LVMI) was calculated as LVM/BSA.

**Figure 2:**
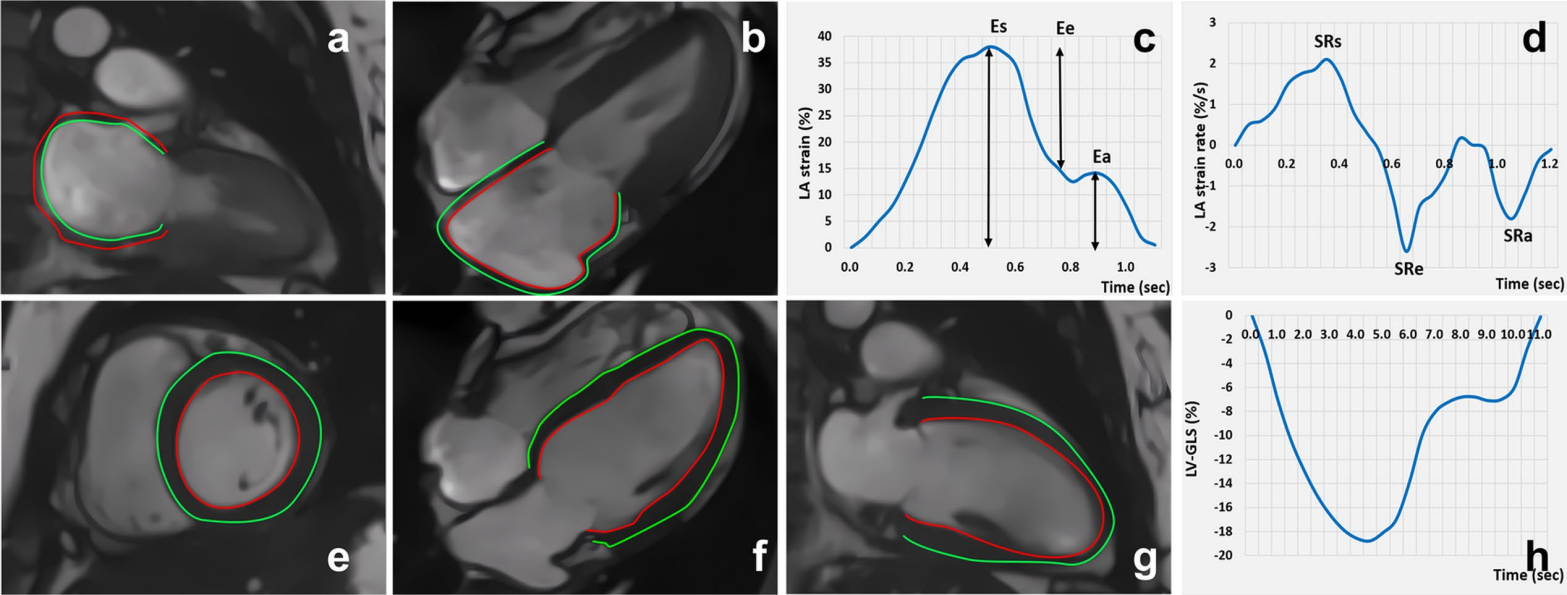
Cardiac magnetic resonance feature tracking and strain curve: LA strain **(A-D)**; LV strain **(E-H)**.

In the cvi42 tissue tracking module, the endocardium and epicardium contours of the LA at the end-diastolic phase of the long-axis two- and four-chamber slice views were manually delineated. The endocardium did not include the LA appendage or the pulmonary vein. The automatic contour tracking algorithm was used to obtain the LA longitudinal strain values, including reservoir strain (LAEs), conduit strain (LAEe), and booster strain (LAEa). The duration of the phase was also calculated. In addition, the LA positive peak strain rate (representing the reservoir strain rate, LA-SRs), LA early negative peak strain rate (representing the conduit strain rate, LA-SRe), and LA late negative peak strain rate (representing the booster strain rate, LA-SRa) were acquired using longitudinal strain rate curves (Figures 2A–D).

Similarly, in the cvi42 tissue tracking module, the endocardium and epicardium contours of the LV were automatically delineated in the cine images at the end-diastole phase from the short-axis and long-axis two- and four-chamber slice views. The strain and strain rate parameters of the LV including the global strain (LV GLS, LV global longitudinal strain; LV GRS, LV global radial strain; and LV GCS, LV global circumferential strain), segmental strain (LS-apical/mid/basal, LV longitudinal strain at the apical, mid, and basal levels; RS-apical/mid/basal, LV radial strain at the apical, mid, and basal levels; and CS-apical/mid/basal, LV circumferential strain at the apical, mid, and basal levels), and the peak systolic strain rate (PSSR-L/R/S) and peak diastolic strain rate (PDSR-L/R/S) of the longitudinal, radial, and circumferential strains were obtained (Figures 2E–H).

### 2.4 Intra-observer and inter-observer reproducibility of the LA and LV myocardial strain parameters

A total of 50 individuals, including 10 controls, 20 patients with a comorbid burden <2, and 20 patients with a comorbid burden ≥2, were randomly selected. The LA and LV myocardial strain in these participants was measured by two observers to evaluate intra- and inter-observer variability. On two independent measurements, 1 month apart, one observer assessed the same set of participants to evaluate intra-observer variability. To determine inter-observer variability, the second observer assessed the same set of participants 2 months later. During the variability assessment, each observer was blinded to the individuals’ status and the findings of the other observer.

### 2.5 Statistical analysis

SPSS 20.0 (IBM-SPSS, Armonk, New York) and R language software packages were used for data analyses. The Shapiro–Wilk test was performed to evaluate the normality of the distribution of continuous variables. The normally distributed continuous variables were reported as means ± standard deviations (SD), while the non-normally distributed variables were expressed as medians and interquartile ranges. Categorical data were presented as frequencies (percentage), and chi-squared test and Fisher’s exact test were used to assess the between-group differences. One-way analysis of variance (ANOVA), when the data followed a normal distribution, and the Kruskal–Wallis test, when the data showed a skewed distribution, were used to compare the baseline clinical characteristics among the control, comorbid burden <2, and comorbid burden≥2 groups. After adjusting for age, sex, and body mass index (BMI), analysis of covariance (ANCOVA) was performed to assess the differences between the three groups with respect to CMR-derived geometry and strain parameters. The Bonferroni *post-hoc* test was used for pairwise comparisons. In all univariate analyses, *p*-values of <0.05 were considered statistically significant.

We constructed two combined prediction models using the comorbid burden and clinical baseline data for the early prediction of LV GLS-reflected LV myocardial function damage (Model 1) and LAEs-reflected LA myocardial function damage (Model 2). There was considerable variability in the strain values measured using the different methods (STE or CMR-FT), vendors, and software packages. This study synthesized several studies (15–17) on normal strain values and defined LV myocardial function damage as LV GLS <16% and LA myocardial function damage as LAEs <32%. These criteria were used for secondary grouping. To screen for predictors, univariable logistic regression was performed to assess the relationship between the LV GLS or LAEs as a dependent variable and comorbid burden and the clinical baseline indicators as independent variables. The predictive parameters with a *p*-value of <0.05 in the univariate logistic analysis were included in the backward multivariate logistic regression model to identify the independent factors influencing LV myocardial dysfunction and LA myocardial function decline. Multivariable prediction probability was used for receiver operator characteristic (ROC) curve analysis. Bootstrap sampling was used for the internal validation of the predictive models. In the ROC curve analysis, the area under the curve (AUC) from 1,000 repeated samples was used to evaluate the predictive performance of the models.

The inter- and intra-observer agreements were assessed by determining intraclass correlation coefficients (ICCs).

## 3 Results

### 3.1 Clinical baseline characteristics

The clinical baseline characteristics of the individuals in the control, comorbid burden<2, and comorbid burden≥2 groups are compared in Table 1. The comorbid burden≥2 group had higher BMI and BSA levels than the control group (*p* < 0.001 for both). The uric acid (UA) levels in the comorbid burden <2 and comorbid burden≥2 groups were significantly higher than those in the control group (*p* < 0.001). The medication history of patients with comorbid burden is presented in Table 2. Notably, there were no significant differences in the proportion of the patients receiving antihypertensive, antidiabetic, and statin therapy between the comorbid burden <2 and comorbid burden≥2 groups.

**Table 1:** Clinical baseline characteristics of the study cohort.

	Controls (*n* = 82)	Comorbid burden <2 (*n* = 97)	Comorbid burden ≥2 (*n* = 101)	*p*-value
Demographics				
Age (years)	50.65 ± 9.10 (95% CI: 48.65, 52.65)	55.55 ± 11.72* (95% CI: 53.39, 57.71)	55.03 ± 10.08* (95% CI: 53.04, 57.02)	0.002
Height (m)	1.69 ± 0.08	1.69 ± 0.08	1.71 ± 0.07	0.046
Weight (kg)	69.95 ± 14.58 (95% CI: 66.74, 73.15)	73.89 ± 14.06 (95% CI: 71.05, 76.72)	79.13 ± 15.08*^#^ (95% CI: 76.15, 82.11)	<0.001
BMI (kg/m^2^)	24.39 ± 3.57 (95% CI: 23.61, 25.17)	25.62 ± 3.56 (95% CI: 24.90, 26.34)	26.83 ± 4.07* (95% CI: 26.03, 27.64)	<0.001
BSA (m^2^)	1.75 ± 0.23 (95% CI: 1.70, 1.80)	1.81 ± 0.21 (95% CI: 1.787, 1.85)	1.89 ± 0.22*^#^ (95% CI: 1.84, 1.93)	<0.001
Male, sex; *n* (%)	40 (48.8%)	57 (58.8%)	62 (61.4%)	0.205
Smoking; *n* (%)	12 (14.6%)	22 (22.7%)	20 (19.8%)	0.392
Drinking; *n* (%)	5 (6.1%)	9 (9.3%)	9 (8.9%)	0.705
Medication		43 (44.3%)	51 (50.5%)	0.385
Hemodynamic variables				
SBP (mmHg)	123.90 ± 16.26 (95% CI: 120.33, 127.47)	136.27 ± 20.78* (95% CI: 132.08, 140.46)	144.52 ± 20.10*^#^ (95% CI: 140.56, 148.49)	<0.001
DBP (mmHg)	82.95 ± 10.33 (95% CI: 80.68, 85.22)	88.58 ± 12.47* (95% CI: 86.06, 91.09)	93.44 ± 13.66*^#^ (95% CI: 90.74, 96.13)	<0.001
HR (bpm)	72.21 ± 7.47	74.00 ± 11.20	75.64 ± 9.99*	0.063
Laboratory data				
HbA1c (%)	5.40 (0.40)	5.60 (0.40) *	5.80 (1.10) *^#^	<0.001
PBG (mmol/L)	6.65 (2.13)	7.98 (3.63) *	10.0 (4.41) *^#^	<0.001
FBG (mmol/L)	5.25 (0.50)	5.47 (0.95) *	5.74 (1.25) *	<0.001
LDL-C (mmol/L)	2.78 ± 0.63 (95% CI: 2.65, 2.92)	3.19 ± 0.83* (95% CI: 3.02, 3.36)	3.08 ± 1.04 (95% CI: 2.88, 3.29)	0.006
HDL-C (mmol/L)	1.38 ± 0.32 (95% CI: 1.31, 1.45)	1.31 ± 0.32 (95% CI: 1.25, 1.38)	1.16 ± 0.29*^#^ (95% CI: 1.11, 1.22)	<0.001
TC (mmol/L)	4.81 ± 0.73 (95% CI: 4.65, 4.97)	5.32 ± 1.04* (95% CI: 5.11, 5.53)	5.33 ± 1.21* (95% CI: 5.09, 5.57)	<0.001
TG (mmol/L)	0.99 (0.59)	1.41 (0.80) *	2.16 (1.65) *^#^	<0.001
eGFR (ml/min)				
≥90	75 (91.5%)	83 (85.6%)	87 (86.1%)	0.432
60–90	7 (8.5%)	14 (14.4%)	14 (13.9%)	
UA (umol/L)	322.84 ± 92.76	379.43 ± 98.05*	386.29 ± 101.86*	<0.001
	(95% CI: 302.46, 343.22)	(95% CI: 359.67, 399.19)	(95% CI: 366.18, 406.40)	

Data are presented as mean ± standard deviation, frequency (percentage), or median (interquartile range).

*p < 0.05 versus controls, ^#^*p* < 0.05 versus comorbid burden<2.

BMI, body mass index, calculated as weight(kg)/height(m)^2^; BSA, body surface area, calculated as 0.006*height; (cm) + 0.0128*weight(kg)-0.153; SBP, systolic blood pressure; DBP, diastolic blood pressure; HR, heart rate; HbA1c, glycated hemoglobin; PBG, postprandial blood glucose; FBG, fasting blood glucose; LDL-C, low-density lipoprotein cholesterol; HDL-C, high-density lipoprotein cholesterol; TC, total cholesterol; TG, triglyceride; eGFR, estimated glomerular filtration rate; UA, uric acid.

**Table 2:** Medication history in the comorbid burden cohort.

	Comorbid burden<2 (*n* = 97)	Comorbid burden ≥2 (*n* = 101)	*p*-value
Antihypertensive medication			
ACEI/ARB; *n* (%)	19 (19.6%)	28 (27.7%)	0.179
Beta-blocker; *n* (%)	10 (10.3%)	6 (5.9%)	0.260
CCB; *n* (%)	43 (44.3%)	34 (33.7%)	0.124
Insulin; *n* (%)	7 (7.2%)	5 (5.0%)	0.504
Antidiabetic medication			
Biguanides; *n* (%)	19 (19.6%)	20 (19.8%)	0.970
α-Glucosidase inhibitor; *n* (%)	19 (19.6%)	23 (22.8%)	0.584
Sulfonylureas; *n* (%)	9 (9.3%)	16 (15.8%)	0.165
SGLT-2 inhibitor; *n* (%)	9 (9.3%)	12 (11.9%)	0.552
GLP-1/DPP-4 inhibitor; *n* (%)	8 (8.2%)	7 (6.9%)	0.726
Lipid-lowering medication			
Statins; *n* (%)	13 (13.4%)	21 (20.8%)	0.168

All values are presented as *n* (%). ACEI/ARB, angiotensin-converting enzyme inhibitor/angiotensin-receptor blocker; CCB, calcium channel blocker; SGLT-2 inhibitor, sodium-glucose co-transporter 2 Inhibitor; GLP-1/DPP-4 inhibitor, glucagon-like peptide-1/dipeptidyl peptidase 4 inhibitor.

### 3.2 CMR-derived LA and LV conventional parameters

As shown in Table 3, after adjusting for age, sex, and BMI, there were no significant differences in the conventional structural (LAV _min_, LAV _max_, LAVI, LVESV, and LVEDV) and functional (LAEF and LVEF) parameters of the LA and LV between the control, comorbid burden<2, and comorbid burden≥2 groups (*p* > 0.05 for all). However, the LVM and LVMI in the comorbid burden≥2 group were significantly higher than those in the control and comorbid burden<2 groups (*p* < 0.001 for all), although the LVM in the three groups was within the normal range (18).

**Table 3:** CMR-derived conventional and strain parameters after adjusting for age, sex, and BMI.

	Controls (*n* = 82)	Comorbid burden<2 (*n* = 97)	Comorbid burden≥2 (*n* = 101)	*p*-value
Conventional parameters				
LAV _min_ (mL)	26.94 ± 1.19	26.95 ± 1.04	28.79 ± 1.04	0.378
LAV _max_ (mL)	59.81 ± 2.08	59.39 ± 1.82	62.37 ± 1.82	0.473
LAEF (%)	55.44 ± 1.12	54.39 ± 0.99	54.57 ± 0.99	0.769
LAVI (mL/m^2^)	33.18 ± 1.17	32.84 ± 1.03	33.94 ± 1.03	0.742
LVESV (mL)	50.95 ± 1.43	52.50 ± 1.26	54.35 ± 1.25	0.218
LVEDV (mL)	134.60 ± 2.74	136.85 ± 2.41	139.32 ± 2.40	0.451
LVEF (%)	62.41 ± 0.67	61.59 ± 0.59	61.29 ± 0.59	0.464
LVM (g)	84.50 ± 2.35 (95% CI: 79.88, 89.12)	87.29 ± 2.06 (95% CI: 83.23, 91.35)	96.10 ± 2.06*^#^ (95% CI: 92.05, 100.15)	<0.001
LVMI (g/m^2^)	45.86 ± 1.13 (95% CI: 43.64, 48.09)	46.68 ± 0.99 (95% CI: 45.73, 49.63)	51.64 ± 0.99*^#^ (95% CI: 49.69, 53.58)	<0.001
Strain parameters				
LAEs (%)	38.08 ± 1.07 (95% CI: 35.98, 40.19)	36.38 ± 0.94 (95% CI: 34.53, 38.23)	33.74 ± 0.94 * (95% CI: 31.90, 35.59)	0.011
LAEa (%)	16.06 ± 0.55	16.80 ± 0.49	15.90 ± 0.49	0.379
LAEe (%)	21.94 ± 0.76 (95% CI: 20.44, 23.45)	19.63 ± 0.67 (95% CI: 18.31, 20.95)	17.42 ± 0.67 * (95% CI: 16.10, 18.74)	<0.001
LA-SRs (1/s)	1.86 ± 0.06 (95% CI: 1.75, 1.98)	1.63 ± 0.05 * (95% CI: 1.53, 1.73)	1.56 ± 0.05 * (95% CI: 1.47, 1.67)	0.001
LA-SRa (1/s)	2.07 ± 0.07	2.19 ± 0.06	2.10 ± 0.06	0.418
LA-SRe (1/s)	2.27 ± 0.08 (95% CI: 2.11, 2.44)	1.96 ± 0.07 * (95% CI: 1.81, 2.10)	1.74 ± 0.07 * (95% CI: 1.59, 1.89)	<0.001
LV GLS (%)	18.66 ± 0.22 (95% CI: 18.22, 19.10)	17.91 ± 0.20 * (95% CI: 17.52, 18.29)	16.48 ± 0.19 *^#^ (95% CI: 16.10, 16.86)	<0.001
LS-basal (%)	20.10 ± 0.38	19.63 ± 0.33	19.22 ± 0.33	0.241
LS-mid (%)	17.01 ± 0.38 (95% CI: 16.26, 17.75)	15.82 ± 0.33 (95% CI: 15.17, 16.48)	15.65 ± 0.33 * (95% CI: 14.99, 16.30)	0.020
LS-apical (%)	18.58 ± 0.40 (95% CI: 17.79, 19.36)	16.69 ± 0.35 * (95% CI: 16.00, 17.39)	16.09 ± 0.35 * (95% CI: 15.40, 16.78)	<0.001
LV GRS (%)	34.88 ± 0.77 (95% CI: 33.36, 36.40)	32.35 ± 0.68 * (95% CI: 31.01, 33.69)	31.78 ± 0.68 * (95% CI: 30.44, 33.12)	0.010
RS-basal (%)	35.90 ± 0.82 (95% CI: 34.29, 37.51)	32.08 ± 0.72 * (95% CI: 30.66, 33.49)	32.31 ± 0.72 * (95% CI: 30.90, 33.72)	<0.001
RS-mid (%)	32.59 ± 0.79	31.04 ± 0.69	30.78 ± 0.69	0.212
RS-apical (%)	41.30 ± 1.31	39.42 ± 1.15	38.88 ± 1.15	0.379
LV GCS (%)	19.74 ± 0.28 (95% CI: 19.18, 20.29)	18.83 ± 0.25 (95% CI: 18.34, 19.32)	18.52 ± 0.25 * (95% CI: 18.04, 19.01)	0.006
CS-basal (%)	19.91 ± 0.29 (95% CI: 19.34, 20.49)	18.63 ± 0.26 * (95% CI: 18.14, 19.15)	18.70 ± 0.26 * (95% CI: 18.17, 19.17)	0.002
CS-mid (%)	19.06 ± 0.30	18.43 ± 0.26	18.31 ± 0.26	0.148
CS-apical (%)	21.47 ± 0.42	20.94 ± 0.37	20.64 ± 0.37	0.343
PSSR-L (1/s)	1.25 ± 0.05	1.12 ± 0.05	1.25 ± 0.04	0.080
PSSR-R (1/s)	1.27 ± 0.05	1.29 ± 0.04	1.17 ± 0.04	0.087
PSSR-C (1/s)	0.90 ± 0.02	0.87 ± 0.02	0.87 ± 0.02	0.444
PDSR-L (1/s)	1.43 ± 0.06 (95% CI: 1.32, 1.54)	1.18 ± 0.05* (95% CI: 1.08, 1.28)	1.27 ± 0.05 (95% CI: 1.17, 1.37)	0.006
PDSR-R (1/s)	1.32 ± 0.06	1.29 ± 0.06	1.13 ± 0.06	0.052
PDSR-C (1/s)	0.94 ± 0.02 (95% CI: 0.90, 0.98)	0.88 ± 0.02 (95% CI: 0.85, 0.92)	0.83 ± 0.02* (95% CI: 0.90, 0.87)	0.001

Data are presented as mean ± standard deviation.

*p < 0.05 versus controls, ^#^*p* < 0.05 versus comorbid burden <2.

LAV _min_, minimum LA volume; LAV _max_, maximum LA volume; LAEF, LA active emptying fraction; LAVI, left atrial volume index; LVESV, left ventricular end-systolic volume; LVEDV, left ventricular end-diastolic volume; LVEF, left ventricular ejection fraction; LVM, LV mass at end-diastole; LVMI, LV mass index; LAEs, LA reservoir strain; LAEa, LA booster strain; LAEe, LA conduit strain; LA-SRs, LA positive peak strain rate; LA-SRa, LA late negative peak strain rate; LA-SRe, LA early negative peak strain rate; LV GLS, LV global longitudinal strain; LS-apical/mid/basal, LV longitudinal strain on the apical, mid, basal; LV GRS, LV global radial strain; RS-apical/mid/basal, LV radial strain on the apical, mid, basal; LV GCS, LV global circumferential strain; CS-apical/mid/basal, LV circumferential strain on the apical, mid, basal; PSSR-L/R/S, peak systolic strain rate in the longitudinal, radial, and circumferential.

PDSR-L/R/S, peak diastolic strain rate in the longitudinal, radial, and circumferential.

### 3.3 CMR-FT-derived LA and LV strain parameters

**Figure 3:**
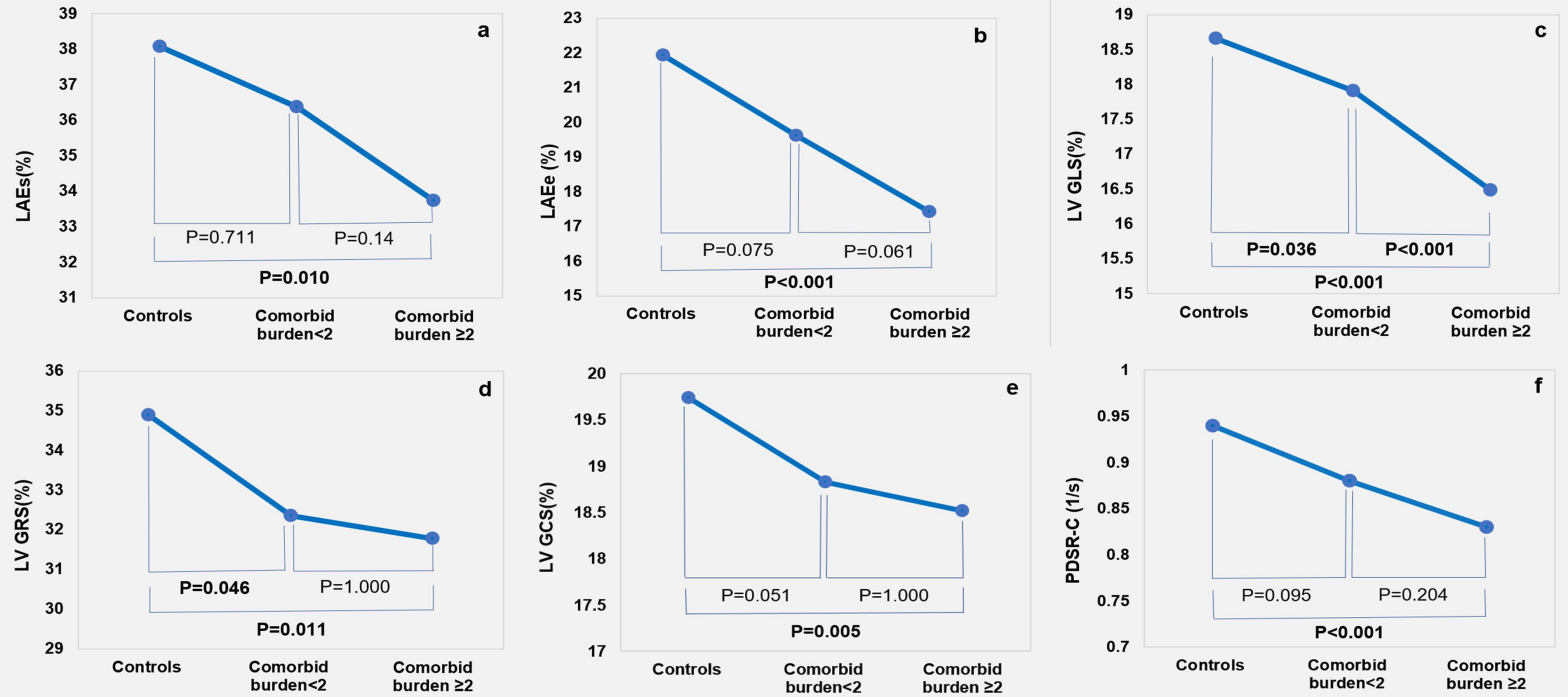
Cardiac magnetic resonance feature tracking findings among the control, comorbid burden<2, and comorbid burden≥2 groups: LA strain **(A,B)**; LV strain **(C-E)**; and circumferential peak diastolic strain rate **(F)**.

As shown in Table 3 and Figure 3, after adjusting for age, sex, and BMI, the LAEs (*p* = 0.011) and LAEe (*p* < 0.001) were lower in the comorbid burden≥2 group than the control group. The LA-SRs (*p* = 0.001) and LA-SRe (*p <* 0.001) were decreased in the comorbid burden<2 and comorbid burden≥2 groups compared to the control group.

The LV GLS (*p* < 0.001) was lower in the comorbid burden≥2 group than in the comorbid burden <2 and control groups, the LV GCS (*p* = 0.006) was significantly lower in the comorbid burden≥2 group but was preserved in the comorbid burden<2 group, and the LV GRS (*p* = 0.010) was lower in the comorbid burden group than in the control group. In addition, we compared the LV segmental strain in three directions and found that the LS-apical (*p*<0.001), LS-mid (*p* = 0.020), RS-basal (*p* < 0.001), and CS-basal (*p* = 0.002) were impaired in the comorbid burden≥2 group. No significant difference was observed between the three groups with respect to the radial, circumferential, and longitudinal PSSR. However, the circumferential PDSR in the comorbid burden≥2 group was significantly lower than that in the control group (*p* = 0.001).

### 3.4 Prediction models for myocardial function damage based on the combination of comorbid burden and the clinical indicators

**Figure 4:**
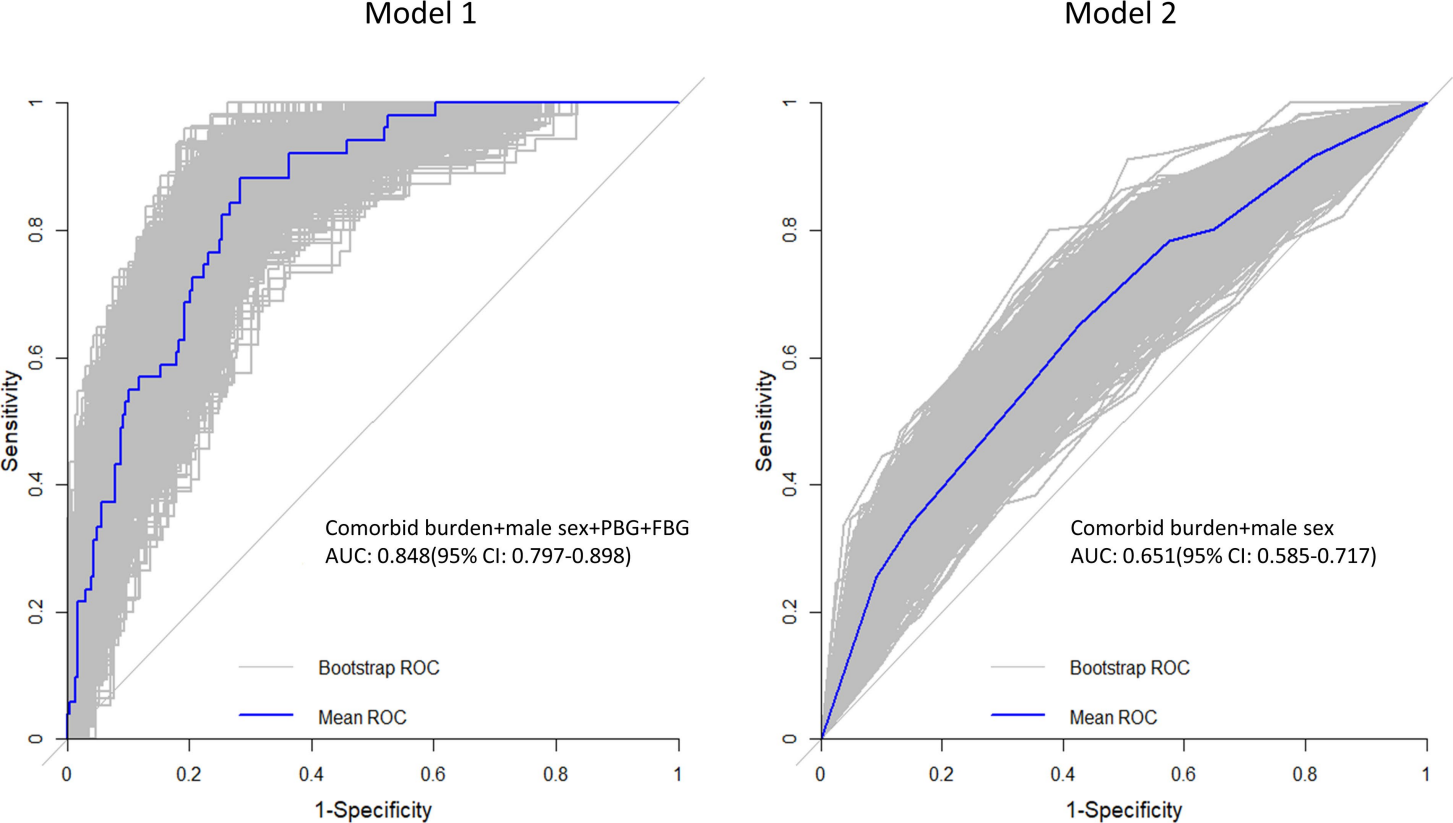
Receiver operating characteristic (ROC) curve. The gray line represents the ROC curve for the 1,000 repeated samples, while the blue line represents the ROC curve for the original data. Comorbid burden combined with the male sex, PBG, and FBG predicts LV myocardial function damage (Model 1), and comorbid burden combined with the male sex predicts LA myocardial function damage (Model 2).

The results of univariate and multivariate logistic regression analyses using the LV GLS or LAEs as a dependent variable and comorbid burden and the clinical baseline indicators (such as BMI and postprandial blood glucose (PBG)) as independent variables are shown in Supplementary Tables S1, S2. The final results (Table 4 and Figure 4) showed that comorbid burden combined with male sex, PBG, and fasting blood glucose (FBG) predicted LV myocardial function damage (mean AUC = 0.848, 95% CI: 0.797, 0.898), and comorbid burden combined with male sex predicted LA myocardial function damage (mean AUC = 0.651, 95% CI: 0.585, 0.717).

**Table 4:** Prediction models for myocardial function damage based on the combination of comorbid burden and the clinical indicators.

	Model 1: LV myocardial dysfunction	Model 2: LA myocardial function decline
Mean AUC	0.848	0.651
*p*-value	<0.001	<0.001
95% CI	0.797–0.898	0.585–0.717

Model 1, comorbid burden combined with the male sex, PBG, and FBG predicts LV myocardial function damage.

Model 2, comorbid burden combined with the male sex predicts LA myocardial function damage.

### 3.5 Intra-observer and inter-observer variability

As shown in Table 5, there was excellent intra-observer (ICC, 0.824–0.966) and inter-observer (ICC, 0.833–0.945) consistency in the measurement of the LA and LV myocardial strain.

**Table 5:** Intra-observer and inter-observer variability of the myocardial strain parameters.

	Intra-observer		Inter-observer	
	ICC	95% CI	ICC	95% CI
LAEs	0.879	0.796–0.930	0.853	0.744–0.916
LAEa	0.824	0.704–0.897	0.833	0.719–0.903
LAEe	0.888	0.811–0.935	0.890	0.814–0.936
GLS	0.892	0.793–0.942	0.869	0.763–0.927
GRS	0.966	0.941–0.981	0.945	0.905–0.968
GCS	0.905	0.836–0.946	0.881	0.799–0.931

## 4 Discussion

This study explored the effects of comorbid burden on left atrial and left ventricular myocardial functions in patients without organic heart disease. First, we demonstrated that the LA reservoir and conduit functions were impaired under comorbid burden, despite the normal traditional parameters of LA function, such as LAEF and LAVI. Second, although the LVEF was preserved, the LV GLS, GRS, and GCS were already impaired in the comorbid burden group, and the LV GLS was the earliest and most severely affected parameter. Third, we found that the LS-apical, LS-mid, RS-basal, and CS-basal were the earliest parameters affected by comorbid burden. Finally, we demonstrated that comorbid burden combined with the male sex, PBG, and FBG can perfectly predict LV myocardial function damage and that comorbid burden combined with the male sex can predict LA myocardial function damage with good accuracy.

Early detection of subclinical cardiac structural and functional abnormalities can help identify asymptomatic individuals who are at risk of adverse cardiovascular outcomes. The LA plays an important role in LV filling and involves three phases: during LV systole, the LA acts as a reservoir for collecting pulmonary venous regurgitation; during early diastole, the LA acts as a passage for blood flow to the LV, a conduit function; and during late diastole, the LA’s booster function acts as a foundation for active LV filling (4, 19). Our results showed that the LA reservoir and conduit functions (LAEs, LAEe, LA-SRs, and LA-SRe) were impaired in the comorbid burden≥2 group, while the volumetric parameters, such as LAV _max_ and LAVI, were normal. In addition, the circumferential PDSR in the patients with a comorbid burden ≥2 and the longitudinal PDSR in the patients with a comorbid burden <2 were also decreased, indicating impairment of LV diastolic function in the presence of comorbid burden (Table 3). Diastolic dysfunction refers to the decreased deformability of the LV due to the impaired ability of the myocardium to relax. Studies have shown that the elevation of LA pressure, resulting from impaired LV diastolic function, is a predominant pathophysiologic process that reduces LA reservoir function (5). LA size is related to LV diastolic function and is a known indicator of long-term exposure to elevated LV filling pressures. However, hypertension and diabetes are associated with impaired LV diastolic function, independent of the effect of overweight/obesity and other covariates (20). In addition, obesity and metabolic syndrome can lead to alterations in myocardial lipid metabolism, an increase in myocardial fat and epicardial fat content, and heightened inflammatory and oxidative stress, eventually leading to cardiac lipotoxicity and diastolic dysfunction (11). In the present study, the patients with a comorbid burden of ≥2 had a normal LAV but the highest BMI. After adjusting for BMI, the LAEs was still significantly impaired in the comorbid burden ≥2 group. This indicates that hypertension, T2DM, and dyslipidemia may have a synergistic effect on impaired LV diastolic function, further leading to LA reservoir function impairment in patients with greater comorbid burden.

It was interesting that the LA booster pump function (LAEa, LA-SRa, and LAEF) was preserved in the comorbid burden groups in our study. This may be attributed to the normal LA volume (LAV _min_, LAV _max_) and LAVI in the patients with comorbid burden because LA booster pump function is influenced by intrinsic atrial contractility and correlates with LA size (4). Numerous studies have indicated that LA myocardial interstitial fibrosis, one the most important pathophysiological substrates for atrial fibrillation development, most commonly occurs in cardiomyopathy. This may affect atrial compliance, further impairing LA reservoir and conduit function. Nevertheless, LA booster pump function is largely unaffected as cardiomyocytes are not replaced by fibrosis (19). Hence, we assumed that myocardial interstitial fibrosis occurs in the LA under comorbid burden but that cardiomyocytes remain in a normal condition.

In conclusion, we demonstrated that evaluation of the myocardial strain can help detect early myocardial function impairment in patients with comorbid burden who do not have organic heart disease, even at a stage when conventional functional parameters of the LA are normal. A similar phenomenon was observed in the LV. The LV myocardial strain is used to measure the contractile function of the heart. During systole, the longitudinal strain represents the longitudinal shortening (from the base to the apex) of subendocardial fibers; the circumferential strain manifests as circumferential shortening in a short-axis view, governed by subepicardial fibers; and the radial strain refers to myocardial deformation toward the center of the LV cavity. All of these contribute to radial thickening (8). In this study, we evaluated the global and segmental LV myocardial strains in patients with comorbid burden to provide a comprehensive assessment of myocardial function damage.

The LV GLS was impaired in both the comorbid burden <2 and comorbid burden ≥2 groups, despite normal LVEF. In addition, the apical and mid longitudinal strains were also impaired in the comorbid burden≥2 group. This suggests that myocardial function damage associated with comorbid burden begins in the subendocardium. Previous studies have shown that electric activation originates from the apical subendocardium and peak longitudinal shortening requires a shorter time to occur at the apex (21). Subendocardial dysfunction can be caused by the majority of progressive myocardial diseases and contribute to the decline in longitudinal systolic function (22). On the one hand, myocardial ischemia, along with interstitial and perivascular fibrosis, tends to primarily affect the subendocardium in hypertension (12). With the concomitant presence of T2DM or dyslipidemia, cardiovascular endothelial cells enter a state of chronic inflammation and microvascular disturbance, leading to aggravation of myocardial dysfunction (10, 11). UA is an end product of purine metabolism in humans and great apes, and it may have deleterious effects on cardiovascular health by increasing oxidative stress or promoting local and systemic inflammation (23). In the present study, UA levels were increased in the patients with comorbid burden, potentially promoting endocardial inflammatory changes in these individuals. On the other hand, the value of the LV longitudinal strain has been reported to be inversely associated with increased blood pressure, and even a slight elevation in BP or afterload may affect longitudinal systolic function (24). Our results showed that SBP and DBP were significantly elevated in the patients with comorbid burden. This may result from the loss of cardiac muscle compliance due to fibrosis caused by elevated blood pressure (22).

With the development of subepicardial myocardial hypertrophy, circumferential mechanics increase to compensate for impaired longitudinal function (22). Consistent with this finding, the patients with a comorbid burden ≥2 in our study showed a significant increase in the LVM but impairment in the GCS and GRS. In addition, the comorbid burden group had significantly higher blood pressure. Hence, we hypothesized that, to maintain normal LVEF, LV remodeling occurred in the patients with comorbid burden due to the impaired longitudinal systolic function and significantly increased blood pressure. LV remodeling is defined as the progressive change in LV structure and geometry, resulting from multiple mechanisms such as myocardial ischemia or fibrosis, usually involving chamber dilation and/or hypertrophy (25). A study showed that fiber shortening along the circumferential strain axis and thickening along the radial strain axis are reduced due to a decrease in the circumferential and radial strains. This leads to an increase in LV cavity volume due to reduced inward displacement of the endocardium (26). However, in the present study, the patients with comorbid burden had normal LVESV and LVEDV, indicating that the patients with comorbid burden had a concentric type of LV remodeling (12). Hypertension, T2DM, and dyslipidemia are closely related to myocardial ischemia or fibrosis, further contributing to LV remodeling (10–12). This study further confirmed the early effect of comorbid burden on the LV structure and function by evaluating the myocardial strain parameters. Notably, although the peak systolic strain rate was lower in the comorbid burden groups than in the control group, no significant difference was found. This suggests that the strain rate has lower sensitivity than the strain itself.

Although the CMR-FT-derived strain and strain rate parameters can enable the assessment of subclinical myocardial function damage at an early stage, they are cumbersome and expensive, thereby preventing their wider clinical application. Therefore, we developed two prediction models for LV and LA myocardial function damage, demonstrating that comorbid burden and the male sex are common factors influencing myocardial dysfunction in the left heart. Epidemiologic studies have suggested that the incidence of cardiovascular disease in premenopausal women is lower than that in age-matched men. In addition, although postmenopausal women have a higher risk of CVD than premenopausal women, the incidence of CVD in postmenopausal women is still lower than that in age-matched men (27). This is attributed, at least in part, to the protective role of estrogen against cardiovascular. Estrogen modulates cardiovascular physiology and function by increasing angiogenesis and vasodilation and decreasing reactive oxygen species, oxidative stress, and fibrosis in both healthy and diseased states (27, 28). In addition, estrogen has potent acute and chronic vasodilator effects that ultimately reduce blood pressure. Estrogen can also reduce lipid accumulation (28). The above evidence explains why the male patients with comorbid burden were found more likely to develop myocardial impairment than their age-matched female counterparts. Previous studies have shown that acute hyperglycemia in asymptomatic diabetic patients induces significant changes in GLS (29). In addition, hyperglycemia can cause capillary rarefaction and pericyte loss, which are accompanied by decreased contractility and increased stiffness. Moreover, the cardiac endothelium in the setting of hyperglycemia is in a chronic inflammatory state (10). The above evidence suggests that hyperglycemia adversely affects LV function. In conclusion, the prediction models for myocardial function damage are expected to aid in the early detection and prevention of myocardial function damage in patients with comorbid burden but without organic heart disease.

Previous studies have suggested that certain antidiabetic drugs, such as sodium–glucose cotransporter 2 (SGLT-2) inhibitors, improve the LV GLS due to their natriuretic and osmotic diuretic effects, which reduce cardiac preload and afterload (30, 31). In addition, antihypertensive treatment has been shown to significantly improve LV GLS (32). Statins play an important role in the prevention of cardiovascular diseases by regulating blood cholesterol levels, particularly through the reduction of LDL-C levels, via the inhibition of cholesterol synthase (33). However, in the present study, medication history had no significant effect on the myocardial strain in the patients with comorbid burden. This may be because the participants were receiving multiple medications according to their disease status, and our results could not exclude the effects of other drugs on cardiac pathophysiology.

There are some limitations in our study that should be acknowledged. First, the sample size in our study was relatively small, which limited the generalizability of our findings to the larger comorbid burden population. Second, this was a single-center, retrospective cross-sectional study. Longitudinal prospective studies are required to obtain more robust results. Third, only internal validation was performed in this study. Future studies should include external validation to evaluate the generalizability of the model. Fourth, the comorbidities in this study only included hypertension, T2DM, and dyslipidemia, while other comorbidities, such as hyperuricemia and hyperthyroidism, can also adversely affect cardiac function. Future studies should include patients with more comorbidities. Fifth, due to the thin atrial wall and the presence of the LA appendage and pulmonary veins, performing LA tracking with CMR-FT presented several challenges. Finally, there are significant differences in the reference values for the LA and LV myocardial strains across vendors and software packages. As a result, there is a lack of guidelines specifying normal values.

## 5 Conclusion

CMR-FT can be used to assess the early signs of left-sided myocardial function damage in patients with comorbid burden but without organic heart disease, before a decrease in LVEF occurs. Comorbid burden combined with the male sex, PBG, and FBG showed a strong predictive effect on LV myocardial function damage, while comorbid burden combined with the male sex exhibited a good predictive effect on LV myocardial function damage.

## Data Availability

The raw data supporting the conclusions of this article will be made available by the authors, without undue reservation.

## Glossary

LA: left atrial
LV: left ventricular
LAVI: left atrial volume index
LAV max: maximum LA volume
LAV min: minimum LA volume
LAEF: LA active emptying fraction
LVEDV: left ventricular end-diastolic volume
LVESV: left ventricular end-systolic volume
LVM: LV mass at end-diastole
LVMI: LV mass index
CMR-FT: cardiac magnetic resonance feature tracking
b-SSFP: balanced standard steady-state free precession
BMI: body mass index
BSA: body surface area
LV GLS: LV global longitudinal strain
LV GRS: LV global radial strain
LV GCS: LV global circumferential strain
LS-apical/mid/basal: LV longitudinal strain on the apical, mid, and basal
RS-apical/mid/basal: LV radial strain on the apical, mid, and basal
CS-apical/mid/basal: LV circumferential strain on the apical, mid, and basal
PSSR-L/R/S: peak systolic strain rate in the longitudinal, radial, and circumferential
PDSR-L/R/S: peak diastolic strain rate in the longitudinal, radial, and circumferential
LAEs: LA reservoir strain
LAEe: LA conduit strain
LAEa: LA booster strain
LA-SRs: LA positive peak strain rate
LA-SRe: LA early negative peak strain rate
LA-SRa: LA late negative peak strain rate
