# Supplementary material for: Impact of comorbid burden on global left cardiac function and prediction models for myocardial function damage: a cardiac magnetic resonance feature-tracking study
Source: Front Med (Lausanne). 2025 Feb 13;12:1525334. doi: 10.3389/fmed.2025.1525334 (PMC11881595; doi:10.3389/fmed.2025.1525334)
Supplement: Supplementary file 1 [file Table_1.docx]

**Supplementary Data**

**1. Supplementary Tables:**

**Table S1. Univariable logistic regressions assess the relationship between LV GLS or LAEs as a dependent variable and comorbidity, clinical baseline indicators (such as BMI, PBG, et al) as independent variables.**

|  | Model 1: LV myocardial dysfunction (GLS＜or≥16%） | | Model 2: LA myocardial function decline（LAEs＜or≥32%） | |
| --- | --- | --- | --- | --- |
|  | P value | OR (95% CI) | P value | OR (95% CI) |
| Comorbid burden | **＜0.001** | 3.124(2.225-4.388) | **0.002** | 1.422(1.144-1.767) |
| SBP | **0.008** | 1.019(1.005-1.034) | 0.071 | 1.011(0.999-1.023) |
| DBP | **0.005** | 1.033(1.010-1.057) | **0.019** | 1.023(1.004-1.042) |
| HR | 0.185 | 1.021(0.990-1.052) | **0.046** | 1.026(1.000-1.051) |
| Age | 0.513 | 0.990 (0.961-1.020) | 0.753 | 0.996(0.973-1.020) |
| Male sex | **0.014** | 2.318(1.189-4.519) | **0.002** | 2.264(1.363-3.759) |
| BMI | **0.003** | 1.120(1.038-1.209) | **0.015** | 1.082(1.016-1.154) |
| HbA1c | **0.001** | 1.484(1.168-1.884) | 0.085 | 1.218(0.973-1.524) |
| PBG | **0.007** | 1.104(1.028-1.186) | 0.195 | 1.043(0.979-1.111) |
| FBG | **0.001** | 1.283(1.102-1.494) | 0.080 | 1.136(0.985-1.311) |
| LDLC | 0.314 | 0.834(0.586-1.187) | 0.647 | 0.937(0.710-1.237) |
| HDLC | **0.013** | 0.238(0.077-0.736) | **0.049** | 0.448(0.201-0.997) |
| TC | 0.716 | 1.055(0.791-1.406) | 0.417 | 0.909(0.721-1.145) |
| TG | **＜0.001** | 1.801(1.371-2.366) | 0.075 | 1.187(0.983-1.433) |
| UA | **＜0.001** | 1.005(1.002-1.009) | **0.023** | 1.003(1.000-1.005) |
| eGFR | 0.078 | 1.008(0.999-1.017) | 0.975 | 1.000(0.993-1.007) |
| ACEI/ARB | 0.069 | 1.962(0.948,4.061) | 0.946 | 1.023(0.537,1.949) |
| Beta-blocker | 0.472 | 0.650(0.201,0.210) | 0.126 | 2.214(0.799,6.132) |
| CCB | 0.087 | 1.751(0.922,3.323) | 0.289 | 1.336(0.782,2.280) |
| Insulin | 0.536 | 1.528(0.399,5.855) | 0.146 | 2.390(0.739,7.732) |
| Biguanides | 0.689 | 1.188(0.511,2.764) | 0.933 | 1.030(0.514,2.066) |
| α-Glucosidase inhibitor | 0.150 | 1.756(0.816,3.783) | 0.704 | 1.138(0.583,2.224) |
| Sulfonylureas | 0.435 | 1.474(0.557,3.898) | 0.291 | 0.613(0.247,1.520) |
| SGLT-2 inhibitor | 0.492 | 1.447(0.505,4.150) | 0.159 | 1.899(0.778,4.638) |
| GLP-1/DPP-4 inhibitor | 0.854 | 0.885(0.240,3.257) | 0.211 | 1.948(0.685,5.535) |
| Statins | 0.188 | 1.749(0.762,0.402) | 0.961 | 1.018(0.487,2.131) |

**Table S2. Backward multivariate logistic regression screened the** **independent indicators of LV myocardial function damage and LA myocardial function damage.**

|  | Model 1: LV myocardial function damage（GLS＜or≥ 16%） | | Model 2: LA myocardial function damage（LAEs＜or≥32%） | |
| --- | --- | --- | --- | --- |
|  | P value | OR (95% CI) | P value | OR (95% CI) |
| Comorbid burden | ＜0.001 | 3.363(2.312-4.893) | 0.004 | 1.390(1.114-1.734) |
| Male sex | 0.053 | 2.126(0.991-4.559) | 0.004 | 2.147(1.283-3.595) |
| PBG | 0.048 | 0.858(0.738-0.999) |  |  |
| FBG | 0.027 | 1.406(1.040-1.901) |  |  |
